# SNPs Analysis Indicates Non-Uniform Origins of Invasive Mussels (*Mytilus galloprovincialis* Lamarck, 1819) on the Southern African Coast

**DOI:** 10.3390/ani14213080

**Published:** 2024-10-25

**Authors:** Anita Poćwierz-Kotus, Christopher D. McQuaid, Marek R. Lipinski, Małgorzata Zbawicka, Roman Wenne

**Affiliations:** 1 Institute of Oceanology Polish Academy of Sciences, Powstanców Warszawy 55, 81-712 Sopot, Poland; rwenne@iopan.pl; 2 Department of Zoology and Entomology, Rhodes University, Grahamstown 6139, Eastern Cape, South Africa; c.mcquaid@ru.ac.za; 3 Department of Ichthyology and Fisheries Science, Rhodes University, Grahamstown 6139, Eastern Cape, South Africa; lipinski@mweb.co.za; 4 South African Institute of Aquatic Biodiversity (SAIAB), Grahamstown 6140, Eastern Cape, South Africa

**Keywords:** invasion, colonization, introgression, population structure, single nucleotide polymorhism

## Abstract

Understanding the origins of invasive species is necessary to manage them and predict their potential for spreading. The mussel genus *Mytilus* forms an important component of coastal ecosystems in the northern and southern hemispheres. *M. galloprovincialis* is an important invasive species globally, first appearing on the South African coast in the 1970s. Studies using nuclear and mitochondrial DNA indicated that the invasion probably originated from the north-east Atlantic. We used fifty-five polymorphic SNPs to genotype mussels from sites across the coast of South Africa with reference samples from the Mediterranean, the Atlantic, and New Zealand to test for possible introgression of the northern and southern taxa. Low levels of genetic differentiation were confirmed, and all samples grouped with reference samples of the Atlantic form of *M. galloprovincialis*, supporting previous studies. The SNP genotyping, however, allowed the detection of some individuals with genotypes typical of the Mediterranean, indicating that introduced populations in South Africa do not have a uniform origin. The initial population introduced to South Africa may have been genetically heterogenous from the start, coming from a region influenced by both the Atlantic and Mediterranean. Alternatively, multiple introductions may have taken place, originating from different regions, specifically North Africa, southern Europe, and the Mediterranean, building up the final heterogeneity.

## Simple Summary

Non-native species that are introduced to new ecosystems and become invasive cause enormous ecological and financial damage. Predicting the future spread of such species in marine systems is extremely difficult given their ability to disperse readily, but can be facilitated by understanding their origins. This is because different genotypes can display important differences in behaviour and physiological tolerances. The mussel *Mytilus* taxa inhibits coastal regions of the Northern and Southern Hemisphere. *Mytilus galloprovincialis* has the greatest ability of all mussels to colonise new geographic regions. In both hemispheres it forms a critical component of rocky shore communities and has become invasive on four continents, including southern Africa, with powerful consequences for the local biota. Earlier genetic analyses indicated that this invasion was believed to have arisen from northern hemisphere Atlantic populations. We used a more sensitive technique to broadly confirm this understanding while showing that, in addition, some individuals showed links to Mediterranean populations. The implication is that either *M. galloprovincialis* colonised South Africa through multiple invasions or that the initial invasive population was already a mixture of forms. This suggests that the species may exhibit a range of responses to contemporary climate change.

## 1. Introduction

Both merchant and tourist shipping has increased considerably, with increasing numbers of ships entering less accessible areas, such as Antarctica and the Arctic. As a result, the exploitation of previously inaccessible or unknown mineral resources, such as oil, natural gas, and minerals, and increasing maritime trade and tourism have accelerated the spread of invasive species to new areas, including South Africa [1,2]. Mussels are an important component of coastal marine ecosystems, and among the most successful invasive marine animals are mytilid mussels, including the genus *Mytilus* and especially *M. galloprovincialis*. In the case of these organisms, their artificial spread has been enhanced by deliberate introduction to new areas for aquaculture. In newly colonized regions, *M. galloprovincialis* can hybridize with local *Mytilus* taxa and can displace other mussel species and sedentary fauna. *M. galloprovincialis* expanded from its native populations in the Mediterranean Sea and on the South European and North African Atlantic coasts to the northern and southern hemispheres, including North America (Pacific coast of USA and Canada [3,4,5,6]); South America (Argentina [7]), (Brazil [8,9]), (Chile [10,11,12]); Asia (China [13,14]), (Korea [15,16]), (Russia [17,18]), (Japan [19,20,21,22]); New Zealand [23,24,25]. In addition, the Atlantic form of *M. galloprovincialis* has been detected in Australia as an admixture of the Mediterranean form and the native species *M. planulatus* Lamarck, 1819 [26,27]. Hybrids of *M. galloprovincialis* and *M. edulis* Linnaeus, 1758, have appeared in ports of Western Europe and Norway [28]. *M. galloprovincialis* was spread not only as a result of direct human activity but also through natural processes, for example, when attached to debris moved by tsunamis [29].

*M. galloprovincialis* in South Africa appeared in the 1970s, presumably introduced in ship ballast water, but was only detected in later years [30], with the earliest detection in Saldanha Bay on the west coast of the country [31]. Breeding for consumption purposes has been carried out in this bay for many years [32]. From this region, *M. galloprovincialis* expanded its range on the coast of South Africa northwards on average 115 km per year to the central coast of Namibia and southwards approximately 25 km per year to the Cape of Good Hope and eastwards to the Indian Ocean coast [33,34]. The southward expansion of its range has also been associated with introductions for aquaculture purposes [31,35]. Currently, it is common on the Atlantic coast of Namibia and from Rocky Point to East London on the Indian Ocean coast, with a total length of approximately 2800 km [36,37]. *M. galloprovincialis* creates beds on hard substrata in the intertidal zone, which modify local environmental conditions such as temperature, desiccation, and food availability for native species in different biogeographic regions as an ecosystem engineer [38,39,40]. Ultimately, it has become an important aquaculture species in South Africa [41,42].

The coast of South Africa is inhabited by two native species of mussels morphologically similar to *Mytilus*: *Choromytilus meridionalis* Krauss, 1848, and *Perna perna* Linnaeus, 1758 [43,44], and one relatively recent invasive species, *Semimytilus algosus* (*S. patagonicus*) Gould, 1850 [45,46]. For this reason, the use of genetic methods was necessary to identify invasive *M. galloprovincialis* confidently. The first genetic studies, based on the comparison of allozyme electrophoresis and the morphology of the population from South Africa, showed that it was more similar to the population of *M. galloprovincialis* from the Mediterranean coast of Spain than to *M. edulis* from Denmark [30]. The use of the polymorphic nuclear DNA marker mac-1 showed similarity of the South African population to the Atlantic *M. galloprovincialis* from Portugal [10]. The use of another, less polymorphic marker, ME 15-16, confirmed the similarity of the frequency of one allele to Atlantic populations from southern Europe, and restriction analysis (RFLP) of the 16S mitochondrial gene showed the presence of haplogroups from the Northern Hemisphere: *M. galloprovincialis* and *M. edulis*. [47]. Moreover, a sample from a mussel farm in Saldanha Bay tested using SNP genotyping grouped with samples of Atlantic *M. galloprovincialis* [48]. Populations of *M. galloprovincialis* introduced to new areas do not show any differentiation [9]. Populations in South Africa have not previously been studied for spatial diversity using SNPs, which are high-resolution markers in population genetics research [49]. Here, we use SNP analysis to determine genetic diversity, structure, and genetic relationships among wild and farmed *M. galloprovincialis* populations sampled across 1600 km of the Atlantic and Indian Ocean coasts of South Africa and to verify their geographical origins.

## 2. Materials and Methods

### 2.1. Sampling, DNA Extraction and Genotyping

**Figure 1:**
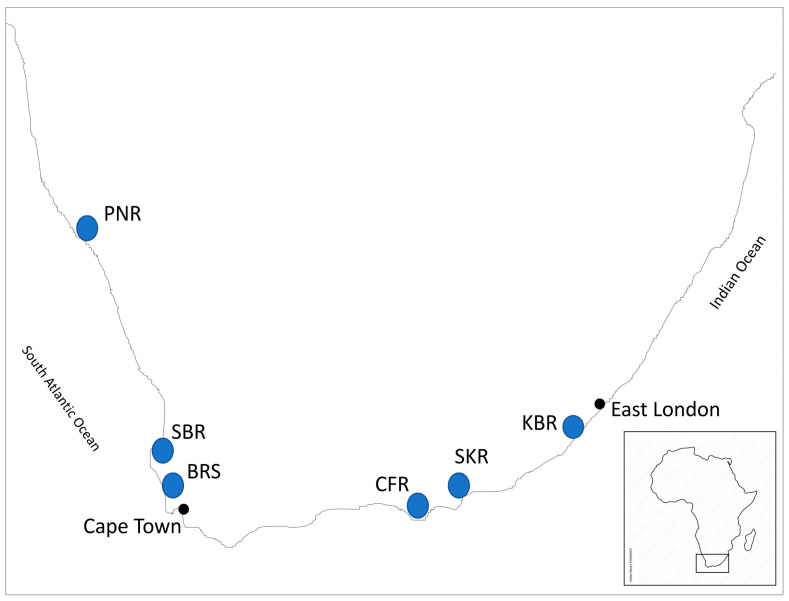
Map of South African *Mytilus galloprovincialis* sample sites in the Atlantic and Indian Oceans. Population codes as shown as Table 1.

Samples of *M. galloprovincialis* were collected from six locations, 5 natural and 1 from a mussel culture site in Saldanha Bay, on the South Atlantic and Indian Ocean coasts of South Africa in 2012. Tissues from 181 individual mussels were preserved in 70% ethanol. Genomic DNA was isolated from the mantle tissue using a modified CTAB method following Hoarau et al. (2002) [50]. DNA genotyping was performed using the Sequenom MassARRAY iPLEX genotyping platform [51] at the Centre for Integrative Genetics (CIGENE) in Norway. Analysis and scoring were performed using Typer 3.4 by Sequenom. Reference samples of *Mytilus* taxa from 24 locations (677 individual mussels with already published genotypes) were used in bioinformatic analyses to determine genetic diversity and to verify the geographic origins of the introduced *M. galloprovincialis* populations in South Africa (Table 1; Figure 1 and Figure S1). These reference samples (677 individuals) included: *M. trossulus* Gould, 1850, from Canada; *M. edulis* from the USA and Great Britain; *M. chilensis* Hupé, 1854, from Chile, and *M. platensis* from Argentina, the Mediterranean, and the Atlantic *M. galloprovincialis*, and *M. planulatus* (*M. aoteanus*) Lamarck, 1819, from New Zealand [27] (Table 1).

### 2.2. Bioinformatic Analyses

To investigate differences in levels of genetic diversity among the six South African *Mytilus* populations and their relationships with the 24 reference populations, the following genetic parameters were calculated using Arlequin v 3.5.1.3 software [53]: number of polymorphic loci (PO), observed (HO), and expected (HE) heterozygosity, inbreeding coefficient FIS, average gene diversity over loci, average number of pairwise differences within the population, and number of loci showing departure from Hardy–Weinberg equilibrium (HWE) (Table S1).

Allele frequencies and minor allele frequency (MAF for bi-allelic) were calculated from spreadsheet data using Arlequin v 3.5.1.3, as well. Arlequin was also used to perform an Analysis of Molecular Variance (AMOVA) to detect variance among the *Mytilus* populations, among populations, within groups, among individuals within populations and within individuals according to different scenarios described in Supplementary Material Table S4 and to estimate the variation within populations by the average number of pairwise differences.

GenAlEx v. 6.5 [54,55] was used to conduct a principal coordinate analysis (PCA) to visualize the relationships between populations by plotting the major patterns within a multivariate dataset. This multivariate technique allowed us to complement the output of the phylogenetic analyses as it is more informative regarding distances among major groups. Genetic structure was estimated using the program STRUCTURE v2.3.4 [56], which assigns individual genotypes to a specified number of groups (K) based on membership coefficients estimated from the genotype data. The analysis for 30 *Mytilus* population samples was conducted from K = 1 to 12 using a burn-in period of 100,000 steps followed by 200,000 MCMC (Monte Carlo Markov Chain) replicates with 5 iterations, assuming an admixture model. The most probable number of clusters was defined by calculating the ΔK value [57] determined by Structure Harvester [58]. Clumpp v.1.1.1 [59] was applied to average cluster membership using the Large K Greedy algorithm. Distruct v.1.1 [60] enabled the visualization of the output from Clumpp. The probability of *Mytilus* populations analysed belonging to reference populations was calculated using the partial Bayesian approach of Rannala and Mountain (1997) [61] implemented in GeneClass version 2.0 [62]. Individuals were considered to be correctly assigned to their location of origin if the assignment probability to that group was higher than any other assignment probability to any other group. A neighbour-joining (NJ) tree illustrating the genetic relationships among populations was constructed on the basis of FST measures in the Newick format, obtained in POPTREEW [63] and visualised in MEGA version 6 [64]. One analysis included all 30 populations, while the second covered 24 *Mytilus galloprovincialis* populations only.

## 3. Results

### 3.1. Analysis of the Genetic Diversity of Mytilus Populations

A total of 181 individuals from 6 South African populations were successfully genotyped using 55 SNP loci (Supplementary Table S1) [52], enabling a calculation of the diversity indices (Supplementary Table S2). The classes of minor allele frequency (MAF) were presented in Figure S2. An average FST computed for South African populations was only 0.001, indicating a low level of differentiation. Genetic diversity is approximately similar in the South African and other populations of *M. galloprovincialis*.

Pairwise comparisons of FST values among all 30 *Mytilus* populations for each locus are detailed in Table S3. 78.6%; all values were significant (*p* < 0.05). Six South African populations vs. the reference populations were genetically differentiated, except for *M. galloprovincialis* populations from the Atlantic (AGA, BID, CAM, CAS, and VIG) and ORAW populations. However, when considering the FST values before Benjamini–Yekutieli [65] correction, statistically significant differences were found between the South African populations PNR and CFR. In comparisons between examined South African populations and the Mediterranean region of *M. galloprovincialis*, the highest FST values were shown for HER, TURK (Aegean Sea), and BLS (Black Sea) populations (averaged FST values for six African populations were 0.1177, 0.1192, and 0.1230, respectively), with the highest values observed for the pairs PNR-HER (1.530), PNR-BLS (0.1562), and PNR-TURK (0.1517). To measure within-population diversity, average pairwise differences were calculated (Table S2). The most diverse of the South African populations was SBR, and the least diverse was PNR. The remaining populations (CFR, KBR, BRS, and SKR) exhibited similar levels of within-population diversity. Analysis of Molecular Variance (AMOVA) was performed comparing groups of samples for six different scenarios where populations were defined a priori (Supplementary Table S4).

### 3.2. Analysis of Genetic Structure and Genetic Relationships Among Populations

**Figure 2:**
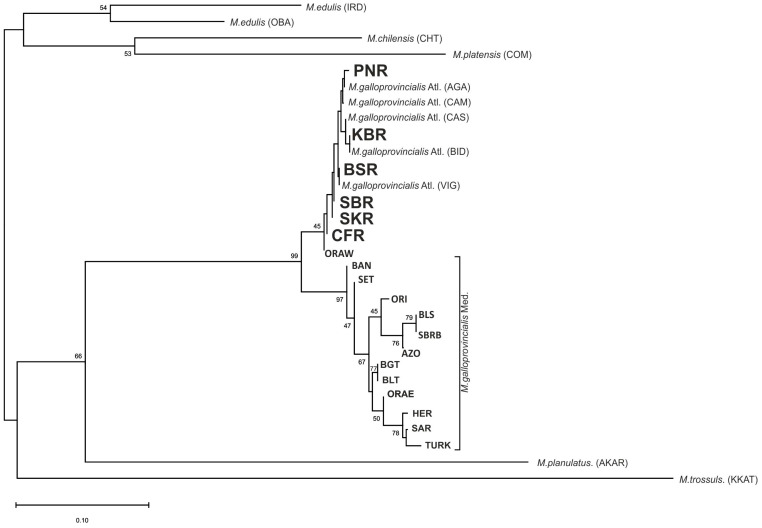
Relationships among 30 *Mytilus* samples including 24 *Mytilus galloprovincialis* samples illustrated by the neighbour joining method. Acronyms of South African populations are in bold. Bootstrap probabilities are shown on the tree. Population codes as shown in Table 1.

Genetic relationships between South African and reference localities were analyzed on the basis of the results obtained from the neighbour-joining (NJ) tree calculated using FST measures (Figure 2). Concerning the analysis of South African populations together with reference populations, the NJ method showed that the analyzed genotypes belonged to five major clades: *M. edulis* (IRD and OBA); *M. chilensis* (CHT) and *M. platensis* (COM); *M. planulatus* (AKAR); *M. trossulus* (KKAT); and *M. galloprovincialis* (containing the six South African populations). A tree topology of *M. galloprovincialis* reflected the placement of South African samples among Atlantic populations. PNR and AGA were found in a common branch of the tree, while KBR was close to the BID and BSR to the VIG. SBR, SKR, and CFR were placed on the separated short branches.

**Figure 3:**
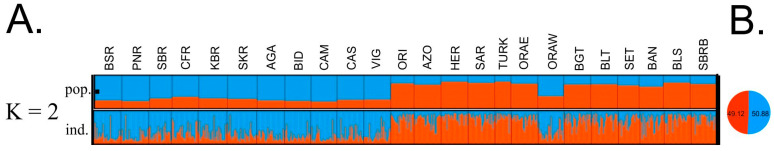
(**A**) Proportion of membership of 693 individuals from 24 *Mytilus galloprovincialis* populations (six South African and 18 references), calculated by Structure v. 2.3.4 software and averaged by Clumpp v. 1.1.1 software. Plots were generated by Distruct v.1.1 software. (**B**) The percentage of genetic variations contained by each genetic group. Population codes as shown in Table 1.

To examine the genetic relationships within the six South African and 18 reference populations of *M. galloprovincialis* species, a structure analysis was performed for these 24 populations alone, excluding reference populations of *M. edulis* (IRD, OBA), *M. trossulus* (KKAT), *M. planulatus* (AKAR), *M. chilensis* (CHT), and *M. platensis* (COM) (Figure 3A). Hypothetical population values (K) from 1 to 12 were tested. The maximum value ΔK was for K = 2 with a secondary peak at K = 5. At K = 2, (ΔK = 86.35), six South African populations with *M. galloprovincialis* from Atlantic populations were found to be distinct from *M. galloprovincialis* from Mediterranean populations (Figure 3). The proportions of total genetic variation contained within each group were very similar: 50.88% and 49.12% (Figure 3B). Results of structure analysis for K = 5 are included in Supplementary Materials Figure S3.

**Figure 4:**
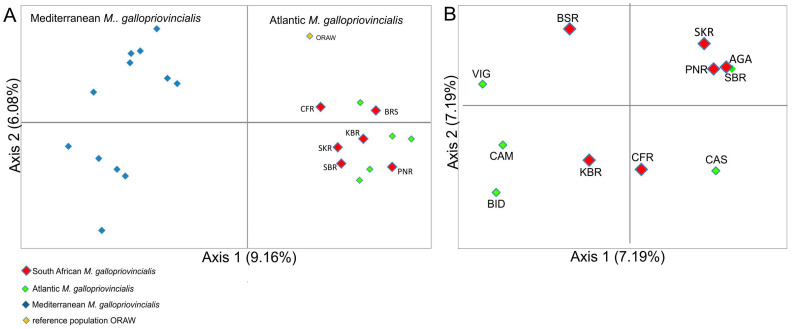
PCA plot for 24 populations showing the two first principal components. (**A**) Principal component 1 (PC1), explaining 9.16% of the variation (Eigenvalue = 34.261) separates twelve Mediterranean *M. galloprovincialis* samples from twelve Atlantic and examined South African *M. galloprovincilis* samples. PC2 explains 6.08% of the variation (Eigenvalue = 22.764). (**B**) PCA plot for eleven Atlantic *M. galloprovincialis* populations showing the two top principal components. Principal component 1 (PC1) explains 7.19% of the variation (Eigenvalue = 18.936) and PC2 explains 6.53% of the variation (Eigenvalue = 17.180). Population codes as shown as Table 1.

To further assess relationships among *Mytilus* populations, a principal coordinate analysis (PCA) was performed. In general analysis including all 30 populations showed the South African populations (PNR, SBR, BSR, CFR, SKR, and KBR) to be grouped together with five references Atlantic populations (BID, VIG, CAS, CAM, and AGA) and one Mediterranean (ORAW), while the second cluster consisted of only reference populations from the Mediterranean (ORAE, BGT, BLT, ORI, SAR, HER, TURK, SBRB, BLS, AZO, SET, and BAN). In the PCA, including only the 24 *M. galloprovincialis* populations, the South African populations formed a cluster with Atlantic populations that was well separated from the others by PC1. PC1 explained 9.16% of the variation (Eigenvalue = 34.261) and PC2 explained 6.08% of the variation (Eigenvalue = 22.764) (Figure 4).

**Figure 5:**
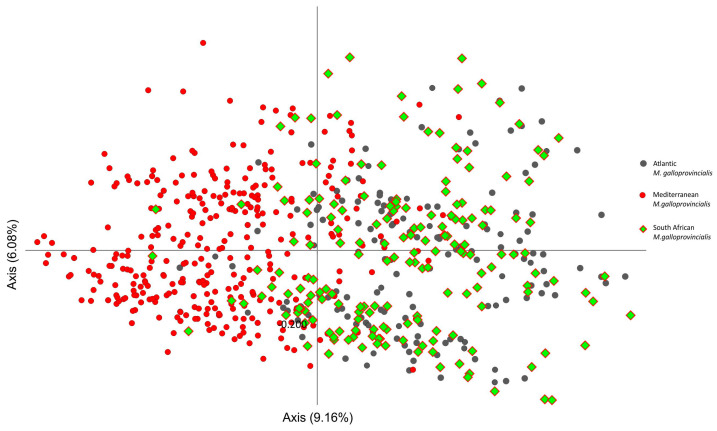
Principal coordinates analysis plot of individual level of stratification for South African populations, Atlantic and Mediterranean reference populations of *M. galloprovincialis*.

Taking into account only the five references Atlantic populations of *M. galloprovincialis*, three of the six South African populations (SKR, PNR, and SBR) were placed quite close to each other and the AGA population (Figure 5). The remaining South African populations (BSR, KBR, and CFR) were more dispersed. Here, PC1 and PC2 explained 7.19% (Eigenvalue = 18.936) and 6.53% (Eigenvalue = 17.180) of the total genetic variation, respectively. The most genetically distant population seems to be the BSR population; this was separated from the other South African populations by PC1, as well as PC2.

PCA analysis (Figure 5) shows the location of individuals from South Africa in relation to individuals from the Atlantic and the Mediterranean Sea. The overlap of South African populations with Atlantic populations is clearly visible. However, the similarity of Mediterranean individuals with some individuals from South Africa is illustrated as well.

An assignment test was carried out with the South African populations assigned to a set of 18 *M. galloprovincialis* populations divided by the origin region: Atlantic, Central Mediterranean, East Mediterranean, and Black Sea. The analysis excluded self-assignment. All reference samples were correctly assigned to their taxa. Most of six South African populations were assigned to the Atlantic form of *M. galloprovincialis* with the highest percentage of assignment being observed for PNR (93.55%) and the lowest for KBR (78.12%) (Table 2). However, a small percentage of individuals were assigned to the Mediterranean forms: 9.37% of KBR individuals and 6.66% of BSR individuals were assigned to *M. galloprovincialis,* from the Central Mediterranean, while 6.66% of CFR individuals and 6.06% of SKR individuals were assigned to *M. galloprovincialis* from the Black Sea. About 15% of the South African individuals were assigned to the Mediterranean and Black Sea populations. In contrast, in the Atlantic reference samples used, this was only the case for about 6%. While only about 2.50% of the individuals from the Mediterranean and Black Seas were assigned to the Atlantic reference populations.

Similarity to the East Mediterranean form of *M. galloprovincialis* was noticeably the lowest among three forms of Mediterranean *M. galloprovincialis* (6.25% of KBR individuals) (Table 2). The close relationship between South African populations and Atlantic *M. galloprovincialis* was supported by a topology of the NJ tree where the PNR sample formed a common cluster with Atlantic AGA and CAM.

## 4. Discussion

In this study, SNP genotyping was used for the first time to investigate genetic polymorphism and the hypothetical geographic origin of *Mytilus galloprovincialis* populations in South Africa. Interregional genetic diversity of *M. galloprovincialis* was analyzed from three locations on the South Atlantic coast and three on the Indian Ocean coast. The tested samples were grouped with reference samples of the Atlantic form of *M. galloprovincialis* in all analyses. This demonstrates their strong similarity, clearly indicating that the source population originated from the Atlantic coast of northern Africa (Morocco) and southern Europe (Spain). These SNP results were generally in agreement with previously published assumptions about the origins of invasive *M. galloprovincialis* populations on the coast of South Africa based on studies using single diagnostic molecular markers: nuclear and mitochondrial DNA [10,47]. This confirmed our existing understanding of this invasion that invasive populations of *M. galloprovincialis* on the coast of southern Africa originated from one or more introductions of the Atlantic form. The use of SNP analysis provides more precise information on within-population diversity [66]; however, it indicated that these populations include individuals with Mediterranean affinities. Although *M. galloprovincialis* from the Atlantic Northeast appears as the most likely source of worldwide exotic settlements instead of the previously thought Mediterranean population [67]. Another possibility for the origin of *M. galloprovincialis* in South Africa is the so-called Dock mussels (admixture between *M. edulis* and the Mediterranean lineage of *M. galloprovincialis*) [28].

Thus, SNP genotyping allowed us to detect individuals with genotypes typical of the inner Mediterranean Sea. This implies that the populations introduced to South Africa do not have a uniform origin, leading to two possible interpretations. First, the mussels originally introduced to an initial site at Saldanha Bay, almost certainly through human intervention [30], may not have been genetically homogeneous, coming from both the Atlantic coast and the Mediterranean Sea (heterogeneity before introduction). The second possible explanation is that multiple introductions took place involving mussels from different geographical regions, including the Atlantic coast of North Africa, southern Europe, and the Mediterranean Sea.

Spatial differentiation of *M. galloprovincialis* populations along the coast of South Africa was very weak, despite the existence of natural barriers to gene exchange among populations. The coast of southern Africa includes three well-defined biogeographic regions, with very clear gradients in seawater characteristics from the cool-temperate, eutrophic upwelling-dominated west coast through the warm-temperate south coast to the oligotrophic sub-tropical east coast [68,69,70]. In addition, there is a potential physical barrier to spread as the Benguela Current, which dominates the west coast, flows from south to north, while the south and especially east coasts are dominated by the Agulhas Current, a western boundary current that flows from the Moçambique Channel in the north-east towards the Cape of Good Hope in the south-west. Together, biogeography and hydrodynamics form potentially strong impediments to spread [36,71,72]. Other examples of weak differentiation exist among both invasive and native species of mussels. Newly established invasive populations of *M. galloprovincialis* in Brazil are also characterised by weak genetic spatial differentiation as assessed with SNP markers [9]. In South Africa, similarly, invasive *Semimytilus algosus* from Chile does not show population differentiation using cytochrome oxidase subunit 1 (COI) sequences of mtDNA on the west coast of South Africa [73]. Likewise, the native mussel *Aulacomya atra* Molina, 1782, does not show genetic differentiation of populations along the western and south-western coasts of South Africa when analysed using mitochondrial (CO1) and nuclear internal transcribed spacer (ITS1) analyses [74]. The most likely explanation for weak divergence in the face of barriers to dispersal lies in mussel life history traits, combined with sufficient continuity of suitable habitat to allow stepping-stone dispersal [75]. Although the mussel *Perna perna* conforms to this broad pattern of panmixia in southern Europe, showing little divergence across 4000 km of coastline [75], in South Africa this species offers a clear counter example. There, native populations of *Perna perna* exist as two distinct genetic lineages that are largely allopatric but overlap across about 200 km of coast [34]. These eastern and western lineages differ genetically at the level of mtDNA, microsatellites, and ITS markers [34,75,76,77] and in both their physiology [78] and behaviour [76]. The case of *P. perna* in South Africa is different; however, the situation clearly reflects secondary contact between two lineages that evolved separately over a protracted period [77].

Low levels of genetic differentiation seem to be characteristic of invasive populations with a relative short history of presence in new localities, as shown here using SNP analysis and by Zardi et al. (2007) [34], who sequenced a 400-bp portion of the South African *M. galloprovincialis* COI mitochondrial DNA gene. Nevertheless, the similarity in levels of SNP polymorphism between South African and native European populations of the *M. galloprovincialis* population indicates no apparent reduction in genetic polymorphism through a founder effect followed by rapid expansion in the new region. Rather, our results indicate multiple introduction events among Atlantic populations with elements from Mediterranean populations prior to the arrival of the species in South Africa. Our identification of individuals with Mediterranean genotypes indicates how different markers can provide different insights into the history of biological invasions.

## 5. Conclusions

We used SNP genotyping with the Sequenom MassARRAY iPLEX platform to investigate the origins of an important invasive species, the mussel *M. galloprovincialis*, on the coast of South Africa. Our study demonstrated that genotypes of South African samples were grouped with reference samples of the Atlantic form of *M. galloprovincialis,* supporting previous studies. However, this methodology also allowed the detection of individuals with genotypes typical of the inner Mediterranean Sea. This indicates that introduced populations in South Africa do not have a uniform origin and that multiple introduction events occurred among Atlantic and Mediterranean populations prior to the arrival of the species in South Africa.

## Figures and Tables

**Table 1:** Location of the 30 samples of *Mytilus*. Sample acronyms: N—number of examined individuals, location, country, water areas, geographic coordinates, and year of sampling of mussel samples. Abbreviations for the South African samples are in bold.

	Sample	N	Location	Country	Water Area	Coordinates		Year
1	BRS	30	Bloubergstrand	South Africa	Atlantic	33°48′47.82″ S	18°27′55.08″ E	2012
2	CFR	30	Cape St. Francis	South Africa	Indian Ocean	34°12′11.43″ S	24°50′21.15″ E	2012
3	KBR	32	Kaysers Beach	South Africa	Indian Ocean	33°15′3.87″ S	27°35′21.70″ E	2012
4	PNR	31	Port Nolloth	South Africa	Atlantic	29°16′7.79″ S	16°51′49.02″ E	2012
5	SBR	25	Saldanha Bay	South Africa	Atlantic	33°1′37.64″ S	18°1′27.32″ E	2012
6	SKR	33	Skoenmakerskop	South Africa	Indian Ocean	34°2′50.62″ S	5°32′42.28″ E	2012
7	AGA ^a^	31	Agadir, Atlantic	Morocco	Atlantic	30°18′3.36″ N	9°48′56.60″ W	2011
8	BID ^a^	29	Bidasoa	Spain	Atlantic	43°21′38.71″ N	1°51′11.15″ W	2004
9	CAM ^a^	29	Camarinal	Spain	Atlantic	36°4′48.01″ N	5°47′58.00″ W	2004
10	CAS ^a^	30	Cascais	Portugal	Atlantic	38°34′14.89″ N	9°19′8.95″ W	2013
11	VIG ^a^	30	Vigo	Spain	Atlantic	42°13′54.12″ N	8°45′7.22″ W	2004
12	AZO ^b^	30	Azov Sea	Ukraine	Azov Sea	45°43′51.71″ N	35°5′0.26″ E	1997
13	BAN ^b^	27	Banyuls, Gulf of Lion	France	Mediterranean	42°27′51.89″ N	3°10′30.49″ E	2004
14	BGT ^b^	30	Bizerta Bay, Gulf of Tunis	Tunisia	Mediterranean	37°16′36.70″ N	9°53′58.20″ E	2013
15	BLS ^b^	30	Crimea	Ukraine	Mediterranean	44°29′0.82″ N	34°12′18.92″ E	2007
16	BLT ^b^	30	Bizerta Lagoon	Tunisia	Mediterranean	37°10′30.89″ N	9°49′41.04″ E	2013
17	HER ^b^	30	Heraklion, Crete, South Aegean S.	Greece	Mediterranean	35°20′40.96″ N	25° 8′56.50″ E	2014
18	ORAE ^b^	30	Oran East, Alboran S.	Algeria	Mediterranean	35°42′36.74″ N	0°39′14.64″ W	2016
19	ORAW ^b^	29	Oran West, Alboran S.	Algeria	Mediterranean	35°10′44.16″ N	1°38′57.67″ W	2016
20	ORI ^b^	29	Oristano	Italy	Mediterranean	39°47′59.88″ N	8°31′9.72″ E	2004
21	SAR ^b^	30	Saronikos Gulf, Aegean S.	Greece	Mediterranean	37°35′39.64″ N	23°16′58.52″ E	2013
22	SBRB ^b^	29	Sunny Beach, South-East Black S.	Bulgaria	Black Sea	42°41′58.74″ N	27°43′3.38″ E	2012
23	SET ^b^	23	Sete, Gulf of Lion	France	Mediterranean	43°23′27.30″ N	3°41′48.11″ E	2013
24	TURK ^b^	18	Izmir, Aegean S.	Turkey	Mediterranean	38° 4′26.33″ N	27°1′19.61″ E	2008
25	IRD ^c^	25	Indian River, Delaware	USA	Atlantic	38°36′27.36″ N	75°3′37.079″ W	2012
26	OBA ^c^	29	Oban, Scottland	Great Britain	Atlantic	6°24′49.40″ N	5°28′23.00″ W	2014
27	KKAT ^d^	28	Halifax	Canada	Atlantic	44°30′33.79″ N	63°29′24.91″ W	1996
28	AKAR ^e^	30	Akaroa South Island	New Zealand	Pacific	43°40′19″ S	172°57′54″ E	2008
29	CHT ^f^	18	Chiloe	Chile	Pacific	42°24′0.54″ S	74°10′48.49″ W	2012
30	COM ^g^	35	Comodoro Rivadavia	Argentina	Atlantic	45°56′00″ S	67°32′0.00″ W	2014

Reference samples—^a^ *M. galloprovincialis* Atlantic: sample 7—[48], 8—[49], 9—[7], 10 and 11—[52]; ^b^ *M. galloprovincialis* Mediterranean: sample 12—[52], 13, 14, 15, 16, 17, 18, 19, 21, 22, 23, 24—[48], 20—[7]; ^c^ *M. edulis*: sample 25—[23], 26—[52]; ^d^ *M trossulus*: sample 27—[7]; ^e^ *M. planulatus*: sample 28—[23,27]; ^f^ *M. chilensis*: sample 29—[9]; ^g^ *M. platensis*: sample 30—[7].

**Table 2:** Assignment testing of South African individuals to reference *M. galloprovincialis* from three locations: Mediterranean and adjacent Black Sea. Tests were computed using GeneClass version 2.0. Population codes as shown in Table 1.

			GeneClass2, Assigned of Individuals to Origin Region						
			Exluding Self-Assignment						
		*M. galloprovincialis*							
		Atlantic		Mediterranean (Central)		Mediterranean (East)		Black Sea	
Name	No ind	No ind	%	No ind	%	No ind	%	No ind	%
BSR	30	25	83.333	2	6.667			3	10.000
PNR	31	29	93.548	1	3.226			1	3.226
SBR	25	22	88.000	3	12.000				
CFR	30	24	80.000	3	10.000	1	3.333	2	6.667
KBR	32	25	78.125	3	9.375	2	6.250	2	6.250
SKR	33	29	87.879	1	3.030	1	3.030	2	6.061

## Data Availability

The data presented in this study will be made available upon request from the authors.

## Supplementary Materials

The following supporting information can be downloaded at: https://www.mdpi.com/article/10.3390/ani14213080/s1. S1. Genetic diversity of *Mytilus* populations, allele frequencies, MAFs, and diversity indices. S2. AMOVA analysis. S3. Structure analysis. Table S1: SNP properties, genome location, references, GenBank annotation, and substitution type. Table S2: Genetic diversity indices calculated for South African (sample symbols marked in bold) and 24 reference *Mytilus* localities. Table S3: FST distance matrix for 55 SNP, 30 *Mytilus* samples. Distance method: pairwise differences. Table S4. Summary of molecular variance (AMOVA) results showing the distribution of variation in genetic diversity of SNP markers among analyzed South African and reference populations calculated for six scenarios (different grouping of samples). All values were significant for *p* < 0.05. Figure S1. A. Map of references samples of *Mytilus galloprovincialis* from Atlantic and Mediterranean basins. B. Map of South African samples of *Mytilus galloprovincialis*. Population codes are shown in Table 1. Figure S2. Allele frequency distribution: 55 SNPs in 181 *Mytilus* individuals from 6 South African locations ranked according to allele frequency. Figure S3. A. Proportion of membership of 693 individuals from 24 *Mytilus galloprovincialis* populations (six South African and 18 references), calculated for K = 2 and for K = 5 by Structure v. 2.3.4 software and averaged by Clumpp v. 1.1.1 software. Plots were generated by Distruct v.1.1 software. B. The percentage of genetic variations contained by each genetic group. Population codes as shown in Table 1.
