# Supplementary material for: SNPs Analysis Indicates Non-Uniform Origins of Invasive Mussels (Mytilus galloprovincialis Lamarck, 1819) on the Southern African Coast
Source: Animals (Basel). 2024 Oct 25;14(21):3080. doi: 10.3390/ani14213080 (PMC11545541; doi:10.3390/ani14213080)
Supplement: Supplementary file 1 [file animals-14-03080-s001.zip › Supplementary Materials_Table S3.pdf]

Supplementary Materials:

Table S3: FST distance matrix for 55 SNP, 30 *Mytilus* samples. Distance method: Pairwise differences.

	BRS	PNR	SBR	CFR	KBR	SKR	IRD	OBA	AGA	BID	CAM	CAS	VIG	ORI	AZO	HER	SAR	TURK	ORAE	ORAW	BGT	BLT	SET	BAN	BLS	SBRB	KKAT	AKAR	CHT	COMO
**BRS**	0																													
**PNR**	0.0047	0.0000																												
**SBR**	-0.0067	0.0088	0.0000																											
**CFR**	0.0021	0.0169	-0.0005	0.0000																										
**KBR**	0.0055	0.0034	0.0104	-0.0028	0.0000																									
**SKR**	-0.0082	-0.0023	-0.0062	0.0020	0.0064	0.0000																								
**IRD**	**0.4389**	**0.4484**	**0.4429**	**0.4514**	**0.4360**	**0.4446**	0.0000																							
**OBA**	**0.3917**	**0.3956**	**0.3853**	**0.3927**	**0.3834**	**0.3894**	**0.2648**	0.0000																						
**AGA**	-0.0061	0.0027	-0.0015	0.0110	0.0084	0.0084	0.4603	**0.4116**	0.0000																					
**BID**	0.0076	0.0168	0.0056	0.0027	-0.0012	0.0110	0.4038	**0.3495**	**0.0136**	0.0000																				
**CAM**	0.0049	0.0061	0.0082	0.0124	0.0024	0.0138	0.4171	**0.3690**	**-0.0016**	0.0012	0.0000																			
**CAS**	0.0018	-0.0028	0.0000	-0.0032	-0.0038	-0.0027	0.4350	**0.3811**	**0.0062**	-0.0045	0.0016	0.0000																		
**VIG**	-0.0109	0.0014	0.0015	0.0021	-0.0015	-0.0078	0.4190	**0.3769**	**0.0013**	0.0084	0.0013	-0.0027	0.0000																	
**ORI**	**0.0914**	**0.1220**	**0.0812**	**0.0718**	**0.0985**	**0.0722**	**0.5304**	**0.4836**	**0.1068**	**0.1119**	**0.1242**	**0.1080**	**0.0865**	0.0000																
**AZO**	**0.1041**	**0.1308**	**0.0931**	**0.0758**	**0.0978**	**0.0755**	**0.5120**	**0.4653**	**0.1310**	**0.1077**	**0.1327**	**0.0988**	**0.0865**	**0.0278**	0.0000															
**HER**	**0.1237**	**0.1530**	**0.1101**	**0.1003**	**0.1222**	**0.0970**	**0.5256**	**0.4680**	**0.1354**	**0.1318**	**0.1502**	**0.1343**	**0.1123**	0.0329	**0.0558**	0.0000														
**SAR**	**0.1146**	**0.1284**	**0.0907**	**0.0859**	**0.1004**	**0.0773**	**0.5261**	**0.4666**	**0.1232**	**0.1181**	**0.1339**	**0.1153**	**0.1014**	**0.0212**	**0.0527**	0.0075	0.0000													
**TURK**	**0.1244**	**0.1517**	**0.1198**	**0.1009**	**0.1194**	**0.0990**	**0.5691**	**0.4938**	**0.1403**	**0.1438**	**0.1637**	**0.1415**	**0.1189**	**0.0427**	**0.0882**	0.0123	0.0083	0.0000												
**ORAE**	**0.0889**	**0.1117**	**0.0812**	**0.0643**	**0.0865**	**0.0655**	**0.5172**	**0.4658**	**0.0903**	**0.1072**	**0.1144**	**0.1004**	**0.0762**	0.0144	**0.0614**	0.0154	0.0117	0.0046	0.0000											
**ORAW**	-0.0014	0.0110	0.0028	-0.0008	0.0060	-0.0070	**0.4653**	**0.4087**	0.0082	**0.0237**	0.0259	0.0079	-0.0007	**0.0527**	**0.0637**	**0.0808**	**0.0622**	**0.0664**	**0.0423**	0.0000										
**BGT**	**0.0820**	**0.1131**	**0.0782**	**0.0678**	**0.0940**	**0.0612**	**0.5098**	**0.4590**	**0.0946**	**0.1179**	**0.1241**	**0.1056**	**0.0741**	0.0078	**0.0418**	0.0247	**0.0258**	0.0201	0.0015	0.0400	0.0000									
**BLT**	**0.0813**	**0.1109**	**0.0753**	**0.0640**	**0.0901**	**0.0598**	**0.5052**	**0.4536**	**0.0925**	**0.1129**	**0.1192**	**0.1018**	**0.0723**	0.0047	**0.0376**	**0.0170**	**0.0178**	0.0140	-0.0032	**0.0391**	-0.0163	0.0000								
**SET**	**0.0704**	**0.1010**	**0.0624**	**0.0431**	**0.0636**	**0.0571**	**0.5084**	**0.4555**	**0.0770**	**0.0809**	**0.0877**	**0.0831**	**0.0561**	0.0146	**0.0550**	**0.0233**	0.0270	**0.0341**	0.0028	**0.0404**	0.0140	0.0093	0.0000							
**BAN**	**0.0525**	**0.0936**	**0.0465**	**0.0341**	**0.0670**	**0.0453**	**0.5103**	**0.4562**	**0.0636**	**0.0771**	**0.0897**	**0.0713**	**0.0502**	0.0057	**0.0320**	**0.0360**	**0.0384**	**0.0394**	0.0121	0.0248	0.0021	0.0008	0.0046	0.0000						
**BLS**	**0.1289**	**0.1562**	**0.1237**	**0.1002**	**0.1252**	**0.1041**	**0.5364**	**0.4849**	**0.1541**	**0.1495**	**0.1664**	**0.1344**	**0.1146**	**0.0187**	0.0074	**0.0654**	**0.0586**	**0.0784**	**0.0583**	**0.0768**	**0.0263**	**0.0248**	**0.0546**	**0.0336**	0.0000					
**SBRB**	**0.0901**	**0.1364**	**0.0932**	**0.0755**	**0.1033**	**0.0821**	**0.5265**	**0.4760**	**0.1220**	**0.1191**	**0.1410**	**0.1090**	**0.0850**	0.0217	0.0033	**0.0639**	**0.0755**	**0.0853**	**0.0574**	**0.0541**	0.0142	0.0148	**0.0453**	0.0098	-0.0088	0.0000				
**KKAT**	**0.7682**	**0.7782**	**0.7673**	**0.7722**	**0.7722**	**0.7699**	**0.7586**	**0.7713**	**0.7763**	**0.7593**	**0.7688**	**0.7728**	**0.7652**	**0.7769**	**0.7825**	**0.7710**	**0.7748**	**0.7797**	**0.7716**	**0.7726**	**0.7672**	**0.7658**	**0.7701**	**0.7715**	**0.7865**	**0.7855**	0.0000			
**AKAR**	**0.5722**	**0.5966**	**0.5710**	**0.5519**	**0.5600**	**0.5591**	**0.7109**	**0.6823**	**0.5778**	**0.5459**	**0.5722**	**0.5678**	**0.5506**	**0.5972**	**0.6042**	**0.5961**	**0.5947**	**0.6765**	**0.5732**	**0.5707**	**0.5579**	**0.5543**	**0.5860**	**0.5776**	**0.6050**	**0.5925**	**0.8469**	0.0000		
**CHT**	**0.5089**	**0.5425**	**0.5135**	**0.5170**	**0.5191**	**0.5126**	**0.5054**	**0.5295**	**0.5253**	**0.5053**	**0.5119**	**0.5295**	**0.4910**	**0.5245**	**0.5275**	**0.5139**	**0.5301**	**0.5649**	**0.5086**	**0.5202**	**0.4899**	**0.4861**	**0.5047**	**0.5087**	**0.5348**	**0.5199**	**0.7566**	**0.6716**	0.0000	
**COMO**	**0.6064**	**0.6199**	**0.6066**	**0.6098**	**0.6105**	**0.6019**	**0.3843**	**0.5190**	**0.6190**	**0.5925**	**0.5967**	**0.6119**	**0.5899**	**0.6357**	**0.6346**	**0.6272**	**0.6309**	**0.6793**	**0.6195**	**0.6192**	**0.6104**	**0.6059**	**0.6219**	**0.6266**	**0.6440**	**0.6415**	**0.7860**	**0.7605**	**0.3996**	0.0000
