# Supplementary material for: SNPs Analysis Indicates Non-Uniform Origins of Invasive Mussels (Mytilus galloprovincialis Lamarck, 1819) on the Southern African Coast
Source: Animals (Basel). 2024 Oct 25;14(21):3080. doi: 10.3390/ani14213080 (PMC11545541; doi:10.3390/ani14213080)
Supplement: Supplementary file 1 [file animals-14-03080-s001.zip › Supplementary_02.pdf]

Supplementary Data

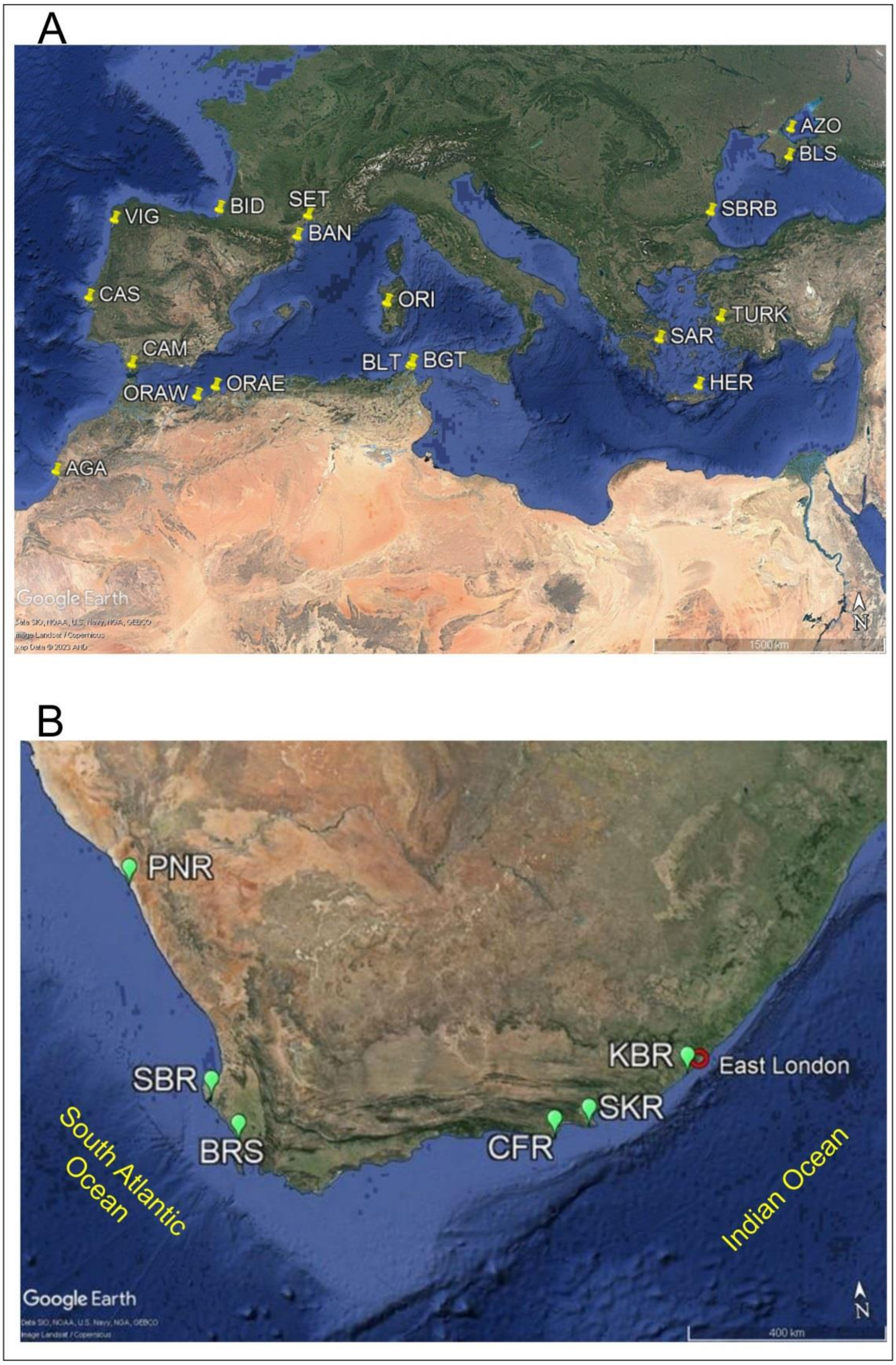

Figure S1 A. Map of references samples of *Mytilus galloprovincialis* from Atlantic and Mediterranean basins. **B.** Map of South African samples of *Mytilus galloprovincialis*. Population codes as shown as Table 1.

S1. Genetic diversity of *Mytilus* populations, allele frequencies, MAFs and diversity indices.

Genotyping analysis was carried out using the Sequenom MassARRAY iPLEX platform. Assays were designed for 79 SNPs selected from 385 putative SNPs based on genotyping quality (positive > 90% of samples). Putative SNPs were designed based on DNA and RNA sequences obtained by us for 16 specimens of *M. trossulus*, *M. edulis* and *M. galloprovincialis* individuals from Europe, North America and sequences obtained from GenBank. Candidate SNPs were selected randomly and tested on 300 specimens of *Mytilus* collected from geographic regions including Europe, North and South America and New Zealand [52], Table S1.

Supplementary Table S1. SNP properties, genome location, references, GenBank annotation and substitution type.

55SNP

	Locus	Location	GenBank annotation	Region	Substitution	Allele	Reference	SNP position in sequence
1	BM101A	Ribosomal protein L7a	KT713378	coding	synon	A/T	Wenne et al. 2016	154
2	BM102A	Proteasome subunit beta type-6-like	KT713379	coding	synon	C/T	Wenne et al. 2016	352
3	BM103B	Proteasome subunit beta type-5-like	KT713380	coding	nonsyn	A/G	Wenne et al. 2016	612
4	BM105A	UnKnown	KT713381	NA	NA	A/G	Wenne et al. 2016	262
5	BM106B	UnKnown	KT713382	NA	NA	A/G	Wenne et al. 2016	154
6	BM10B	Ribosomal protein S20	KJ871040	coding	synon	A/C	Zbawicka et al. 2014	257
7	BM113A	Protein BTG1	KT713385	coding	synon	A/T	Wenne et al. 2016	391
8	BM118A	UnKnown	KT713388	NA	NA	A/G	Wenne et al. 2016	202
9	BM11A	Ribosomal protein L22	KJ871041	coding	synon	A/G	Zbawicka et al. 2014	206
10	BM121A	Ribosomal protein L30	KT713389	coding	synon	A/T	Wenne et al. 2020	162
11	BM12A	Ribosomal protein L23a	KJ871042	coding	synon	C/T	Zbawicka et al. 2014	305
12	BM12C	Ribosomal protein L23a	KJ871042	coding	synon	C/T	Wenne et al. 2016	398
13	BM147A	UnKnown	KT713383	NA	NA	C/T	Wenne et al. 2016	118
14	BM151A	adhesive foot protein gene M.galloprovincialis	HQ257471	coding	synon	G/T	Gardner et al. 2016	51
15	BM16B	ribosomal protein L21	KJ871044	coding	synon	C/T	Wenne et al. 2016	231
16	BM17B	Ribosomal protein L7a	KJ871045	coding	synon	A/G	Zbawicka et al. 2014	330
17	BM201B	*H4 histone* gene	AY267750.1	noncoding	NA	A/C	Zbawicka et al. 2012	167
18	BM201C	*H4 histone* gene	AY267750.1	coding	synon	G/T	Zbawicka et al. 2012	329
19	BM202A	*H3 histone* gene	AY267749.1	noncoding	NA	A/C	Zbawicka et al. 2012	147
20	BM202B	*H3 histone* gene	AY267749.1	coding	synon	A/T	Zbawicka et al. 2012	522
21	BM203B	*H2B histone* gene	AY267742.1	noncoding	NA	C/T	Zbawicka et al. 2012	161
22	BM203C	*H2B histone* gene	AY267742.1	noncoding	NA	C/T	Zbawicka et al. 2012	140
23	BM203D	*H2A histone* gene	AY267757.1	noncoding	NA	A/T	Zbawicka et al. 2012	531
24	BM204A	*p53*	DQ865151	coding	synon	C/T	Zbawicka et al. 2012	411
25	BM21B	qm-like protein	KJ871047	coding	nonsyn	C/G	Zbawicka et al. 2014	211
26	BM21C	qm-like protein	KJ871047	coding	synon	A/C/T	Zbawicka et al. 2014	383
27	BM26B	UnKnown13	KJ871050	NA	NA	A/T	Zbawicka et al. 2014	669
28	BM2G	UnKnown05	KJ871032	coding	synon	G/T	Zbawicka et al. 2014	401
29	BM30A	Ribosomal protein l17	KJ871052	coding	synon	A/G	Zbawicka et al. 2014	146
30	BM30C	Ribosomal protein l17	KJ871052	coding	synon	A/T	Zbawicka et al. 2014	284
31	BM32A	ubiquinol-cytochrome c reductase subunit 6	KT713371	coding	synon	A/G	Wenne et al. 2016	299
32	BM33B	Cytochrome c oxidase subunit IV	KJ871054	coding	synon	A/T	Zbawicka et al. 2014	399
33	BM35C	Ribosomal protein L7	KJ871055	coding	synon	A/T	Wenne et al. 2016	558
34	BM35D	Ribosomal protein L7	KJ871055	coding	synon	A/G	Zbawicka et al. 2014	609
35	BM36F	ribosomal protein S3a	KT713373	coding	synon	A/C	Wenne et al. 2016	419
36	BM38B	ribosomal protein S8e	KT713368	coding	synon	A/G	Wenne et al. 2016	304
37	BM44B	ubiquitin/ribosomal protein S27a	KJ871057	coding	synon	A/G	Zbawicka et al. 2014	274
38	BM50B	CoA-binding protein	KJ871059	coding	synon	A/G	Zbawicka et al. 2014	388
39	BM54A	ETC_C1_NDUFA4	KJ871060	coding	synon	A/G	Zbawicka et al. 2014	179

40	BM57A	NADH-ubiquinone_oxidoreductase	KT713374	coding	nonsyn	C/T	Wenne et al. 2016	334
41	BM57D	NADH-ubiquinone_oxidoreductase	KT713374	coding	synon	A/C	Wenne et al. 2016	164
42	BM5B	Ribosomal protein S6e	KJ871035	coding	synon	A/G	Wenne et al. 2016	288
43	BM5D	Ribosomal protein S6e	KJ871035	coding	synon	C/T	Zbawicka et al. 2014	495
44	BM60A	UnKnown08	KJ871063	coding	synon	A/G	Zbawicka et al. 2014	102
45	BM61A	Ribosomal_L1	KT713375	coding	synon	C/T	Wenne et al. 2016	59
46	BM62A	Ribosomal L13e	KJ871064	coding	synon	A/G	Zbawicka et al. 2014	108
47	BM64A	Ribosomal protein L35	KJ871065	coding	synon	C/T	Zbawicka et al. 2014	295
48	BM67C	Ribosomal protein S6e	KJ871066	coding	synon	A/T	Wenne et al. 2016	375
49	BM6C	EFG_N	KJ871036	coding	synon	C/T	Zbawicka et al. 2014	648
50	BM75C	Nascent polypeptide-associated complex subunit alpha-like	KT713370	coding	synon	C/G	Wenne et al. 2016	56
51	BM78B	UnKnown12	KJ871069	coding	synon	A/G	Zbawicka et al. 2014	376
52	BM8E	Ribosomal protein L3	KJ871038	coding	synon	A/G	Zbawicka et al. 2014	751
53	BM92B	UnKnown06	KJ871074	coding	synon	A/T	Zbawicka et al. 2014	563
54	BM9B	Ribosomal protein S2	KJ871039	coding	synon	A/G	Zbawicka et al. 2014	166
55	BM9C	Ribosomal protein S2	KJ871039	coding	synon	A/C/T	Zbawicka et al. 2014	187

NA, not applicable;

The calculation of the allele frequency enabled the identification of the classes of minor allele frequency (MAF). The mean minor allele frequency amongst polymorphic SNPs was 0.11. Particular classes of MAFs are presented in Figure S2.

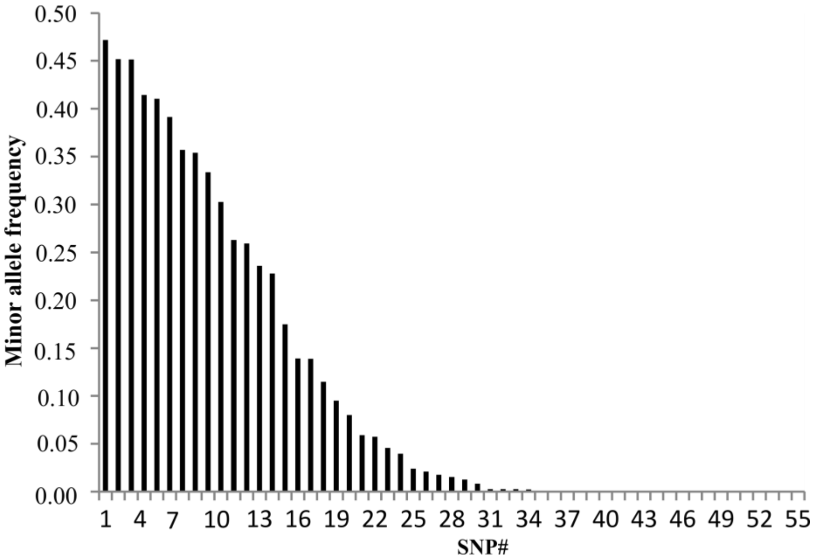

Figure S2. Allele frequency distribution: 55 SNPs in 181 *Mytilus* individuals from 6 South African locations ranked according to allele frequency.

Among examined South African populations, lowest values of polymorphic loci (PO) showed no geographic geographic pattern and were found in populations KBR, PNR and

SBR (49.1%), and the highest in BSR and SKR (54.5%). For 24 reference samples it ranged from 29.1 for *M. planulatus* (AKAR) to 60% for *M. trossulus* (KKAT). For the South African populations, the lowest values of heterozygosity were observed in BSR (HE=0.294; HO=0.228), and the highest in KBR (HE=0.325; HO=0.291), (Table S2). In reference populations HE ranged from 0.147 for *M. planulatus* (AKAR) to 0.336 for Atlantic *M. galloprovincialis* (CAM), and HO ranged from 0.139 in AKAR to 0.327 in Mediterranean *M. galloprovincialis* (ORAE). The numbers of loci deviating from Hardy–Weinberg equilibrium (HWE) after Benjamini–Yekutieli correction [6] for multiple comparisons, were from 0 to 3, demonstrating that the majority of loci were in HWE. For the South African populations, observed heterozygosity (HO) was slightly lower than expected heterozygosity (HE) (differences in values were above 10%) which could indicate a small excess of homozygotes. The greatest difference between HE and HO values occurred at BSR, and the smallest difference at KBR. An average FST computed for South African populations was only 0.001, indicating a low level of differentiation. The global FST across all 30 populations was 0.337. The overall FIS was 0.065 for reference populations, whereas its value 0.121 was higher for the six South African localities .

Supplementary Table S2. Genetic diversity indices calculated for South African (sample symbols marked in bold) and 24 reference *Mytilus* localities.

Sample	PO	HO	HE	MAF	Ad	HWE	FIS
**BSR**	54.545	0.228	0.294	0.110	0.153	3	**0.196**
**CFR**	52.727	0.264	0.302	0.111	0.142	0	0.075
**KBR**	49.091	0.291	0.325	0.111	0.124	1	**0.107**
**PNR**	49.091	0.254	0.303	0.101	0.129	2	**0.145**
**SBR**	49.091	0.261	0.319	0.107	0.137	2	**0.148**
**SKR**	54.545	0.267	0.303	0.113	0.123	0	0.055
AGA	45.455	0.310	0.332	0.104	0.136	0	0.050
BID	50.909	0.317	0.336	0.117	0.157	1	0.060
CAM	47.273	0.316	0.336	0.114	0.142	1	0.071
CAS	50.909	0.273	0.303	0.104	0.144	0	0.053
VIG	54.545	0.277	0.309	0.119	0.152	2	0.073
AZO	49.091	0.274	0.275	0.081	0.133	1	-0.023

BAN	50.909	0.256	0.309	0.101	0.144	3	**0.202**
BGT	54.545	0.283	0.306	0.103	0.133	2	0.065
BLS	49.091	0.289	0.283	0.084	0.124	0	-0.046
BLT	54.545	0.285	0.310	0.105	0.149	2	0.074
HER	52.727	0.272	0.301	0.108	0.140	1	**0.093**
ORAE	50.909	0.327	0.318	0.103	0.139	1	-0.037
ORAW	49.091	0.316	0.317	0.103	0.147	0	0.002
ORI	49.091	0.273	0.287	0.090	0.118	0	0.023
SAR	49.091	0.276	0.302	0.097	0.144	2	**0.097**
SBRB	47.273	0.268	0.297	0.085	0.120	0	0.060
SET	50.909	0.287	0.316	0.111	0.135	1	0.096
TURK	43.636	0.310	0.328	0.090	0.130	0	0.055
IRD	41.818	0.246	0.277	0.077	0.109	0	**0.127**
OBA	49.091	0.206	0.267	0.088	0.114	2	**0.156**
KKAT	60.000	0.199	0.245	0.102	0.113	2	**0.122**
AKAR	29.091	0.139	0.147	0.026	0.034	0	0.015
CHT	36.364	0.302	0.316	0.078	0.134	1	0.017
COMO	32.727	0.229	0.265	0.058	0.074	1	**0.152**

PO, % of polymorphic loci; HO, observed heterozygosity; HE, expected heterozygosity; MAF, minor allele frequency; Ad, Average gene diversity over loci; FIS, inbreeding coefficient; values with $P < 0.05$ after Benjamini–Yekutieli correction are marked in bold. Population codes as shown as Table 1.

S2. AMOVA analysis

Populations were assigned to groups a priori under six different scenarios and analyzed using Analysis of Molecular Variance (AMOVA). Scenario 1 treated all 30 populations as one group, and scenario 2 distinguished eight groups among the 30 populations, dividing them according to species affiliation and location (Table S4). Further scenarios were based on reducing the number of analyzed populations to the 24 populations of *M. galloprovincialis*. Scenario 3 treated all 24 populations as one group. In scenario 4, the 24 populations were divided into two groups: Atlantic and Mediterranean. Scenario 5 included eleven

populations of *M. galloprovincialis* from the Atlantic and South Africa treated as one group whereas scenario 6 included only the six South African populations. Calculated values of the F-statistic were significant for all six scenarios (Table S4). The largest amount of variance was found within individuals for each analyzed model. Compared to scenario 1, with scenario 2, revealed a slight increase in the percentage of variance among groups (from ~35.9 to ~40.1%) and a decrease of variance within individuals (from ~59.2 to ~53.6%). In scenario 2, the resulting variance among populations within groups made only a minor contribution to total variance (1.85%). The contributions to total variance were quite similar when 6 South African populations and 18 reference *Mytilus galloprovincialis* populations were analyzed as either one group in scenario 3, or divided into 2 groups (Atlantic or Mediterranean) in scenario 4. Considering 6 samples of South Africa with Atlantic *M. galloprovincialis* as one group (scenario 5) the percent of variance was predominantly highest within individuals (~90.65%), with small contributions among groups and among individuals within populations. Scenario 6, analyzing only the six South African populations produced a similar result.

Supplementary Table S4. Summary of molecular variance (AMOVA) results showing the distribution of variation in genetic diversity of SNPs markers among analyzed South African and reference populations calculated for six scenarios (different grouping of samples). All values were significant for P < 0.05.

Scenario	Among groups		Among populations within groups		Among individuals within populations		Within individuals	
	**Variance component**	**% variation**	**Variance component**	**% variation**	**Variance component**	**% variation**	**Variance component**	**% variation**
1 - All 30 populations /1 group/	1.78	35.86	--	--	0.240	4.95	2.930	59.19
2 - All 30 populations /8 groups/	2.195	40.08	0.10136	1.85	0.245	4.48	2.934	53.58
3 - *M. galloprovincialis* only /24 pop/1 group/	0.210	5.90	--	--	0.162	4.56	3.190	89.54
4 - *M. galloprovincialis* only /24 pop/2 groups/	0.295	7.98	0.056	1.52	0.162	4.38	3.190	86.12
5 - Atlantic and South Africa *M. galloprovincialis* only /11 pop/1 group/	0.004	0.11	--	--	0.314	9.24	3.076	90.65
6 – South African only *M. galloprovincialis*/6 pop/1 group	0.003	0.09	--	--	0.313	9.42	3.008	90.49

Scenario 1 - All 30 populations /1 group/ = 30 populations (pooled BRS, PNR, SBR, CFR, KBR, SKR, IRD, OBA, AGA, BID, CAM, CAS, VIG, ORI, AZO, HER, SAR, TURK, ORAE, ORAW, BGT, BLT, SET, BAN, BLS, SBRB, KKAT , AKAR , CHT, COM).
Scenario 2 - All 30 populations /8 groups/= South African *M. galloprovincialis* (pooled BRS, PNR, SBR, CFR, KBR, SKR); *M. edulis* (pooled IRD, OBA); Atlantic *M. galloprovincialis* (pooled AGA, BID, CAM, CAS, VIG); Mediterranean Sea *M. galloprovincialis* (pooled ORI, AZO, HER, SAR, TURK, ORAE, ORAW, BGT, BLT, SET, BAN, BLS, SBRB); *M. trossulus* (KKAT); *M. planulatus* (AKAR); *M. chilensis* (CHT); *M. platensis* (COM).
Scenario 3 - *M. galloprovincialis* only /24 populations/1 group/= (pooled BRS, PNR, SBR, CFR, KBR, SKR, AGA, BID, CAM, CAS, VIG, ORI, AZO, HER, SAR, TURK, ORAE, ORAW, BGT, BLT, SET, BAN, BLS, SBRB).
Scenario 4 - *M. galloprovincialis* only /24 populations/2 groups/ = South African and Atlantic *M. galloprovincialis* (pooled BRS, PNR, SBR, CFR, KBR, SKR, AGA, BID, CAM, CAS, VIG); Mediterranean Sea *M. galloprovincialis* (pooled ORI, AZO, HER, SAR, TURK, ORAE, ORAW, BGT, BLT, SET, BAN, BLS, SBRB).
Scenario 5 – South African and Atlantic *M. galloprovincialis* only /11 populations/1 group/ = (pooled BRS, PNR, SBR, CFR, KBR, SKR, AGA, BID, CAM, CAS, VIG)
Scenario 6 – South African only *M. galloprovincialis*/6 populations/1 group = (pooled BRS, PNR, SBR, CFR, KBR, SKR).
Population codes as shown as Table 1.

S3. Structure analysis

In K = 5 (ΔK = 12.20) the distinction of clusters identified was not very unambiguous and it can be seen that the South African populations were assigned to two different clusters: (1) BSR, PNR, SBR and SKR with AGA; (2) CFR and KBR with BID, CAM, CAS and VIG (Figure S3A). The close relationships among PNR, SBR and SKR and among CFR and KBR also were illustrated by a PCA analysis, with the ORAW population forming a separate cluster. The proportions of total genetic variation contained by every genetic group were similar: from 17.94 % to 21.52% (Figure 3B).

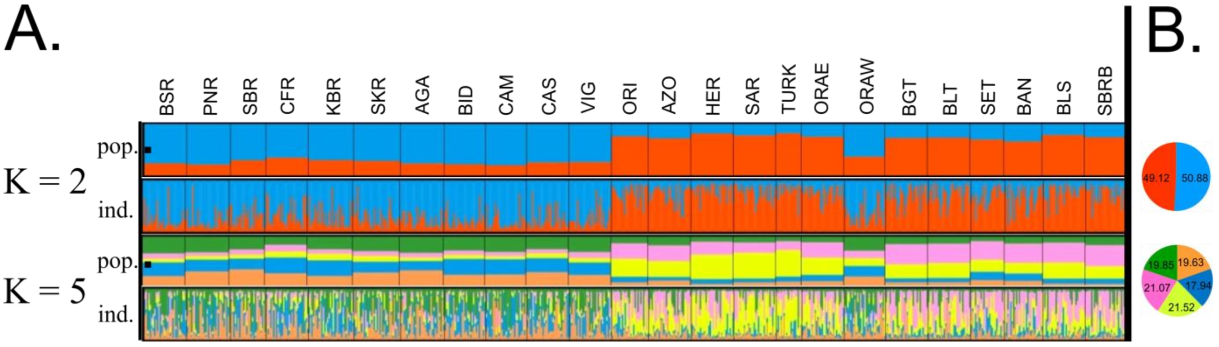

Figure S3. A. Proportion of membership of 693 individuals from 24 *Mytilus galloprovincialis* populations (six South African and 18 references), calculated for K=2 and for K=5 by Structure v. 2.3.4

software and averaged by Clumpp v. 1.1.1 software. Plots were generated by Distruct v.1.1 software. **B**. The percentage of genetic variations contained by each genetic group. Population codes as shown as Table 1.
